# Mushroom poisoning outbreaks in Guizhou Province, China: a prediction study using SARIMA and Prophet models

**DOI:** 10.1038/s41598-023-49095-0

**Published:** 2023-12-18

**Authors:** Li Zhang, Qing-yuan Chen, Su-fang Xiong, Shu Zhu, Ji-gui Tian, Jun Li, Hua Guo

**Affiliations:** 1 Institute of Public Health Surveillance and Evaluation, Guizhou Center for Disease Control and Prevention, Guiyang, 550005 China; 2 Department of Labor Hygiene and Environmental Health, School of Public Health and Wellness, Guizhou Medical University, Guiyang, 550025 China

**Keywords:** Diseases, Health care, Risk factors

## Abstract

Mushroom poisoning is a public health concern worldwide that not only harms the physical and mental health of those who are poisoned but also increases the medical and financial burden on families and society. The present study aimed to describe and analyze the current situations and factors influencing mushroom poisoning outbreaks in Guizhou province, Southwest China, between January 2012 and June 2022, and to predict the future trends of its occurrence. Our study provides a basis for the rational formulation of prevention and control and medical resource allocation policies for mushroom poisoning. The epidemiological characteristics and factors influencing mushroom poisoning incidence were analyzed using descriptive epidemiological methods and the chi-squared test, respectively. Then, future occurrence trends were predicted using the SARIMA and Prophet models. In total, 1577 mushroom poisoning incidents were recorded in Guizhou Province, with 7347 exposures, 5497 cases, 3654 hospitalizations, and 93 fatalities. The mortality rate was 4.45% in 1 ~ 6 years higher than other age groups. There were notable geographic and seasonal characteristics, with the number of occurrences much higher in rural areas (1198) than in cities (379), and poisoning cases were more common during the rainy season (June to September). The mortality rate of household poisoning cases was 1.86%, with the most deaths occurring in households. Statistically significant differences were observed in the incidence across various cities, periods, and poisoning locations (*P* < 0.05). Both models had advantages and disadvantages for prediction. Nevertheless, the SARIMA model had better overall prediction results than the Prophet model (R > 0.9, the residual plot of the prediction results was randomly distributed, and RMSE_SARIMA_ < RMSE_Prophet_). However, the prediction result plot of the Prophet model was more explanatory than the SARIMA model and could visualize overall and seasonal trends. Both models predicted that the prevalence of mushroom poisoning would continue to increase in the future; however, the number of fatalities is generally declining. Seasonal patterns indicated that a high number of deaths from gooseberry mushroom poisoning occurred in October. The epidemiological trends of mushroom poisoning remain severe, and health education on related knowledge must be strengthened in rural areas, with June to October as the key prevention and control phase. Further, medical treatment of mushroom poisoning cases with clinical symptoms should pay attention to inquiries to check whether the mushroom is similar in appearance to the *Amanita*, particularly in October.

## Introduction

According to the *National Foodborne Disease Surveillance Workbook*, a foodborne disease outbreak can be determined when there are at least two illnesses or at least one death in a foodborne disease event. Mushroom poisoning outbreaks are one of the types of foodborne disease outbreaks^1^. Wild mushrooms are becoming increasingly popular owing to their abundant nutrition and unique flavor^2^. Nevertheless, wild edible and poisonous mushrooms have similar forms and appearance; therefore, it is often difficult to distinguish between the two by the naked eye alone. As a result, mushroom poisoning owing to accidental ingestion frequently occurs^3^.

Mushroom poisoning has gradually become a serious public health concern worldwide. It not only imposes a heavy economic and financial burden on the population and government but also increases the risk to people’s lives and health^4^. Depending on the local geography, climatic conditions, and food culture, the epidemiological characteristics of mushroom poisoning frequently vary^5^. In western Iran, 283 patients suffered from cyclopeptide mushroom poisoning in 2018 alone. Among them, 59.01% were rural residents, and ultimately, eight patients died. The total medical cost of these patients was $1,259,349.26^6^. Although the mortality rate of mushroom poisoning is as high as 22%, its frequency is very low (0.1%) in the northeastern Iranian province of Mashhad^7^. In Germany, up to 10,000 cases of mushroom poisoning are reported every year, and the trend is increasing^8^. Accidental consumption of mushrooms containing gooseberry toxins accounted for 15.6% of food-related fatalities between 1995 and 2009 in Switzerland^9^. In the United States, the National Poison Data System reported a cumulative total of 133,700 (7428/year) cases of mushroom exposure between 1999 and 2016. Of these, approximately three deaths per year were caused by mushroom poisoning. Nevertheless, on a per capita basis, this is significantly lower than that reported in European countries such as Italy and France^10^. Therefore strengthening research on local mushroom poisoning outbreaks is of great public health importance to localize control measures.

China has higher poisonous mushroom species than countries such as Japan, Iran, and Turkey, whose rates of mushroom poisoning are second only to that of Russia and Eastern European countries. China had the highest reported mortality rate, making things worse^11^. A cumulative total of 10,036 outbreaks, 38,676 illnesses, and 788 deaths were reported in China between 2010 and 2020^12,13^. As per the official Hunan CDC website, the direct economic burden per capita of patients with poisonous mushroom poisoning is 4,136 yuan, accounting for 34.6% of the per capita disposable income of urban residents in Hunan province (11,930 yuan). Severe cases caused by the consumption of highly toxic goose paste directly led to poverty owing to the disease, with treatment costs reaching an average of > 100,000 per person.

Although mushroom poisoning poses a significant risk, most available studies provide retrospective and descriptive analyses of the incidents^4,14,15^. Additionally, there are several studies on foodborne disease predictions^16–20^. However, forecasting patterns of mushroom poisoning events lack precision. In the present study, we aimed to construct the seasonal autoregressive moving average (SARIMA) and Prophet models, forecast the future trends of mushroom poisoning, and provide some prevention and control recommendations based on these models. We believe that our study will provide the local health departments with a foundation for developing strategies for medical treatment and material distribution and prevention and control policies for mushroom poisoning based on the epidemic’s present state, influencing factors, and occurrence trends.

## Materials and methods

### Study setting

The Guizhou province in Southwest China, one of the poorest provinces economically and with a high rate of mushroom poisoning, was chosen as the subject of this study. The number of incidents and poisonings caused by poisonous mushrooms and their toxins in foodborne disease outbreaks reported in the province between 2004 and 2021 placed Guizhou province in the top three provinces in China, with Yunnan as the only other province to rank higher in terms of the number of deaths^14^. The most common and lethal cause of foodborne illness in the region was mushroom poisoning.

### Dataset

This study was approved by the Ethics Committee of the Center for Disease Control and Prevention of Guizhou Province (Number S2022-09).

The “Guizhou Foodborne Disease Outbreak Surveillance Reporting System” was used to collect the study data, which focused on mushroom poisoning outbreaks that the state had expressly reported between January 2012 and June 2022. The data in this study were reviewed and approved by provincial and national staff, and data from investigative reports that did not pass the review will be excluded. Information on the patient's sex, age group and time of onset will be provided.This study's model was constructed using data from 2012 to 2021, and the model was evaluated using data from January to June 2022.

### Research methods

#### Description of epidemiological characteristics

The epidemiological characteristics of the data on toxic mushroom poisoning outbreaks between January 2012 and June 2022 in terms of the number of exposures, cases, hospitalizations, and deaths in the three-interval distribution were first described. The chi-square test was then used to see if the difference in incidence rates between the different subgroups was statistically significant.

$$\text{Mortality rate }=\frac{Subgroups \, correspond \, to \, the \, number \, of \, deaths}{The \, number \, of \, morbidities \, corresponding \, to \, subgroups.}$$

#### Model selection and construction

The basic idea of the ARIMA model is to take the data series formed by a seasonal phenomenon over time as a new random sequence, use the relationship and characteristics of the random sequence to reflect the plasticity of the predicted development of the phenomenon and apply the relationship and characteristics to the corresponding mathematical model, to achieve the purpose of predicting future values based on the past and present values of the time series. The model is divided into nonseasonal and multiplicative seasonal models. For mushroom poisoning events with typical seasonal and trending distribution characteristics, the multiplicative seasonal ARIMA model, or seasonal autoregressive moving average (SARIMA) model, was constructed. A major limitation of the ARIMA model is that it presupposes linearity^21^. In most cases, the nonlinear structure should also be used for time series analysis because linear models do not produce adequate results. Nevertheless, Prophet models could compensate for this limitation^22^. Therefore, we constructed both the SARIMA and Prophet models, both of which targeted the time series data of mushroom poisoning events with both linear and nonlinear characteristics.

The SARIMA model was constructed using SPSS 23.0 software. The six steps involved in the construction of the SARIMA were as follows: time series creation, smoothness and white noise tests, smoothing process, model identification establishment, model testing and evaluation, and model prediction. The Prophet model was built using the Prophet package in R4.2.0.

### Model evaluation

The Ljung–Box (LBQ) test was used to determine whether the series is a white noise series in the SARIMA model. A fitted model was formed if the *P*-value of the white noise test was greater than the significance level (*P* > 0.05), indicating that the series is white noise^21,23,24^. Based on the parameters RMSEA and measured data between January and June 2022, the SARIMA and Prophet models were assessed. All statistical tests were two-sided with a test level *α* of 0.05.

## Results

### Epidemiological characteristics

#### Basic overview

The number of reported mushroom poisoning outbreaks, cases, and hospitalizations from January 2012 to June 2022 all showed an overall upward trend, whereas the number of deaths showed a downward trend. The cumulative number of reported incidents was 1577, of which 7347 people were exposed, 5495 people became sick, 3654 people were hospitalized, and 93 people died.

#### Time distribution

In the Guizhou province, deadly mushroom poisoning outbreaks occur every year. The severest mushroom poisoning cases were reported in 2020, with the highest number of people who were exposed and hospitalized. Overall, 371 occurrences were documented, leading to 1494 exposures, 1208 cases, and 806 hospitalizations. The year 2012 had the most reported deaths, with 30 fatalities (Table 1). Statistically significant differences in the incidence rates of mushroom poisoning outbreaks across years in Guizhou Province (χ^2^ = 914.798, *P* < 0.001).

**Table 1:** Annual distribution of mushroom poisoning outbreaks in Guizhou province between January 2012 and June 2022.

Year	Exposures	Cases	Hospitalizations	Fatalities	Occurrences
2012	207	189	165	30	43
2013	566	166	133	13	30
2014	132	122	109	7	25
2015	190	136	79	2	40
2016	425	364	272	17	89
2017	1078	846	610	5	254
2018	1200	931	639	6	265
2019	905	681	385	3	201
2020	1494	1208	806	8	371
2021	836	630	339	2	199
2022_(June)_	314	224	117	0	60
Total	7347	5497	3654	93	1577

**Figure 1:**
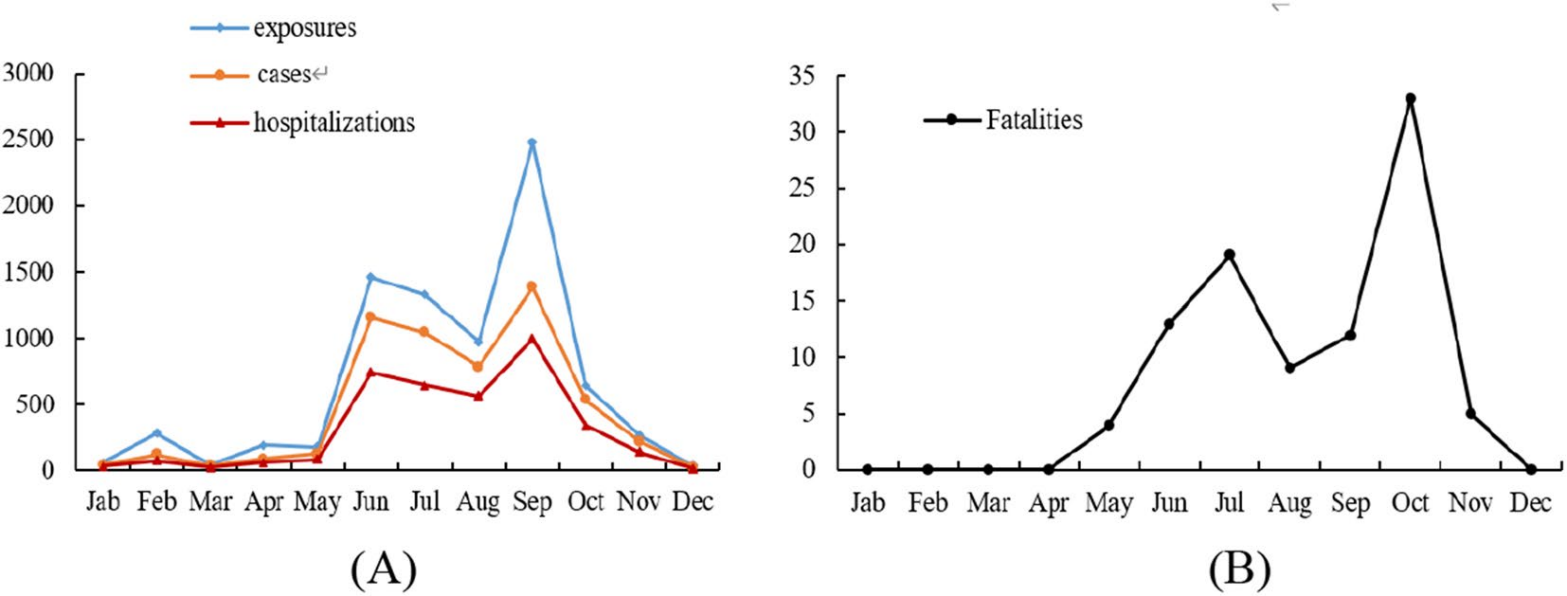
Monthly distribution of mushroom poisoning outbreaks in Guizhou province from January 2012 to June 2022.

In terms of seasonal distribution, there was a detectable seasonal increase in toxic mushroom poisoning outbreaks in Guizhou province, characterized by two peak periods in September and June. With 416 recorded occurrences, 2030 exposures, 1382 cases, 993 hospitalizations, and 12 deaths were reported, making September the month with the most reports, exposures, cases, and hospitalizations. However, October had the most deaths, with 33 deaths, mainly due to the Amanita (Fig. 1). Statistically significant differences in the incidence rates of mushroom poisoning outbreaks in different months in Guizhou Province (χ^2^ = 853.787, *P* < 0.001).

#### Regional distribution

Occurrences of poisonous mushroom poisoning outbreaks were reported in nine cities and states in Guizhou province. Zunyi topped the list in terms of the number of reports, exposures, cases, hospitalizations, and deaths, with 640 reported incidents and 2717 exposures involving 2095 cases, 1423 hospitalizations, and 29 deaths. Tongren was the second city in terms of the number of reports, exposures, cases, and hospitalizations, with 420 reported cases, 1805 exposures, 1364 cases, and 995 hospitalizations. It was also the third-highest city in terms of the number of deaths, with 17 deaths (the second-highest city was in Qiandongnan Autonomous Prefecture). The highest case fatality rate was for Bijie, at 4.44% (Table 2). Statistically significant differences in the incidence rates of mushroom poisoning outbreaks in different regions in Guizhou Province (χ^2^ = 617.547, *P* < 0.001).

**Table 2:** Distribution of mushroom poisoning outbreaks in Guizhou Province from January 2012 to June 2022.

City	Exposures	Cases	Hospitalizations	Fatalities	Occurrences
Anshun	201	159	125	0	39
Bijie	223	180	128	8	44
Guiyang	338	246	143	1	63
Liupanshui	414	320	88	6	88
Qiandongnan	666	569	355	18	151
Qiannan	772	370	259	10	83
Qianxi’na	211	194	138	4	49
Tongren	1805	1364	995	17	420
Zunyi	2717	2095	1423	29	640
Total	7347	5497	3654	93	1577

#### Population distribution

Mushroom poisoning outbreaks were investigated in every age group in Guizhou province from January 2012 to June 2022. The highest number of reported incidents, cases, hospitalizations, and deaths were observed among people aged between 20 and 59 years, with 1358, 3685, 2349, and 51, respectively. The highest case fatality rate was observed among people aged 1 ~ 6 years, with 4.45% (Table 3).

**Table 3:** Population distribution of mushroom poisoning outbreaks in Guizhou Province from January 2012 to 2022.

Age	Occurrences	Cases	Hospitalizations	Fatalities	Case fatality rate
< 1 years	4	5	4	0	0
1 ~ 6 years	219	292	212	13	4.45
7 ~ 19 years	461	674	480	6	0.89
20 ~ 59 years	1358	3685	2349	51	1.38
60 + years	562	841	609	23	2.73
Total	–	5497	3654	93	–

There are multiple ages for one event, so the number of events will overlap.

#### Distribution of disease sites

The locations where deadly mushroom outbreaks occurred in Guizhou province had various traits. A total of 1481 events involving 6036 family-centered individual exposures were documented. These incidents led to 4892 illnesses, 3275 hospitalizations, and 91 fatalities. With a rate of 1.86%, the fatality rate of household cases was likewise the highest across all sites. In addition, 1198 documented occurrences resulted in a total of 5055 exposures, 4086 cases, 2842 hospitalizations, and 91 fatalities, with rural areas reporting the highest case fatality rate (Table 4). Statistically significant differences in the incidence rates of mushroom poisoning outbreaks in different sites in Guizhou Province (χ^2^ = 2996.120, *P* < 0.001).

**Table 4:** Distribution of the places of mushroom poisoning outbreaks in Guizhou province from January 2012 to June 2022.

Location starts	Occurrences	Exposures	Cases	Hospitalizations	Fatalities
Unit canteen	28	322	215	145	0
Family	1481	6036	4892	3275	91
Street stall	10	83	41	12	0
Rural banquet	2	110	23	5	0
Others	40	645	231	145	2
Mega restaurant	9	97	64	59	0
Small restaurant	7	54	31	13	0
City	379	2292	1411	812	2
Rural	1198	5055	4086	2842	91
Total	1577	7347	5497	3654	93

### SARIMA model

#### Model construction

**Figure 2:**
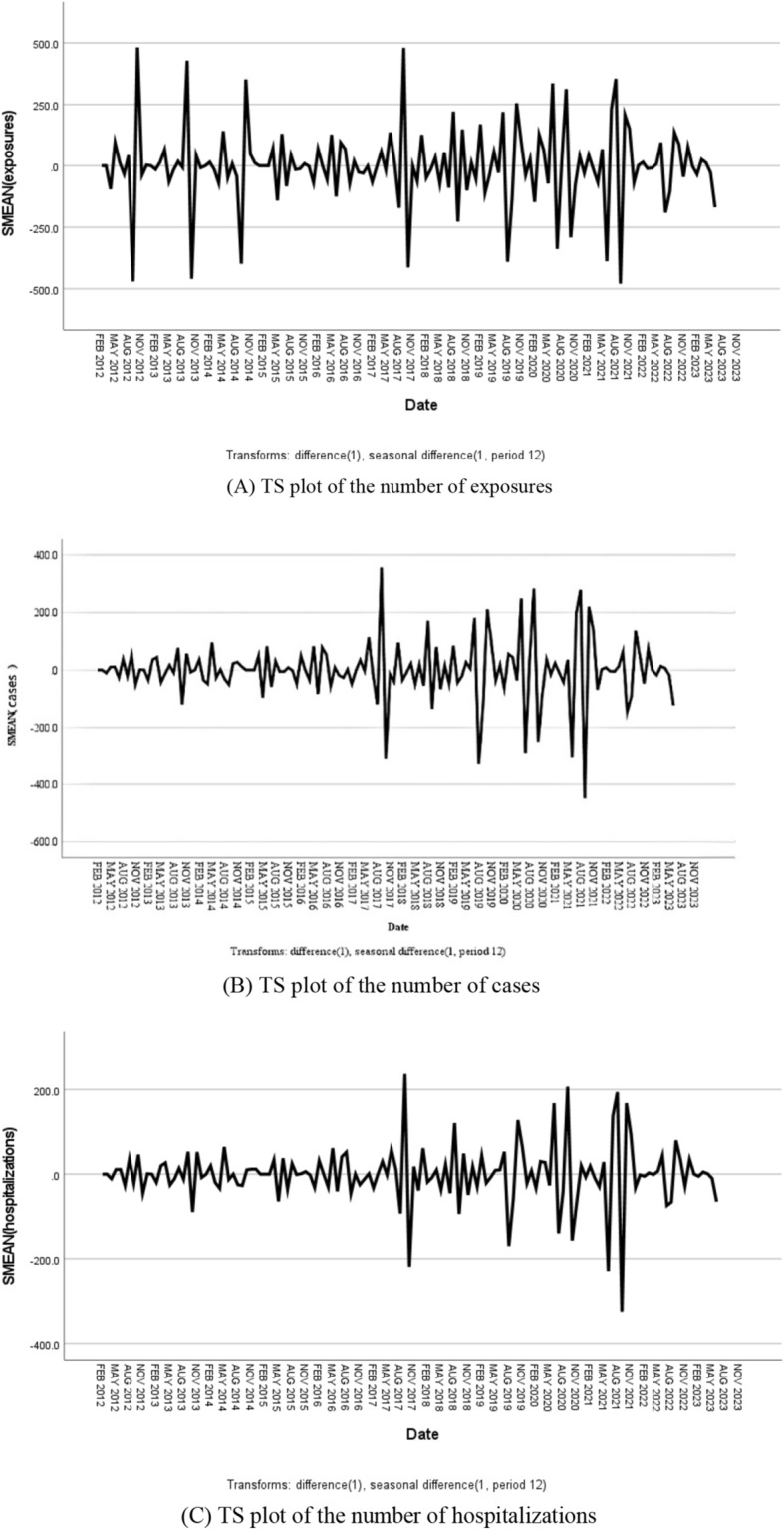
TS plots.

**Figure 3:**
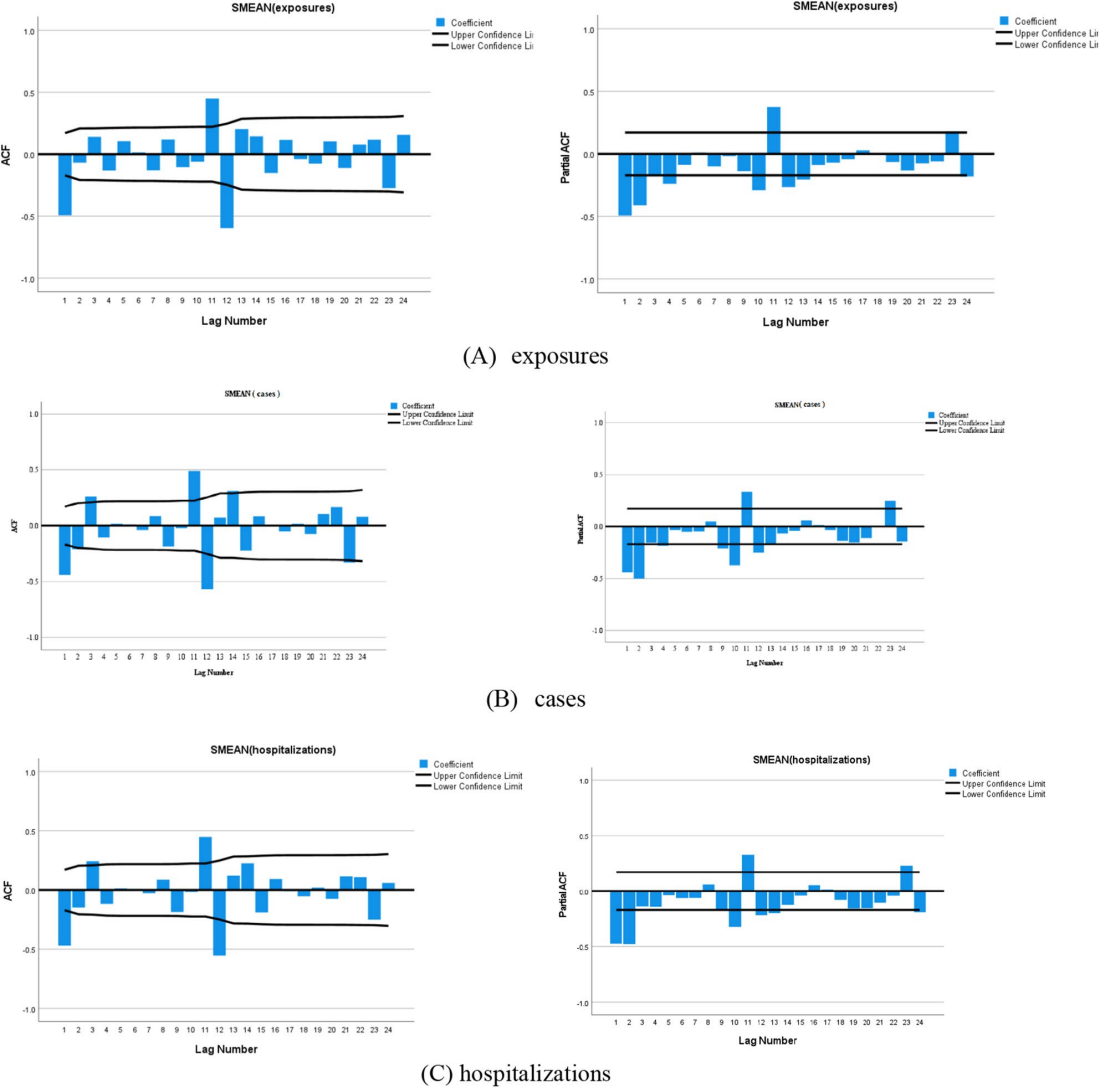
AFC and PAFC plots after first-order difference and first-order seasonal difference.

As the number of fatalities in this study was a white noise series, they were not investigated further. The number of exposures, cases, and hospitalizations were all non-stationary and nonwhite noise series, and all series were smooth after first-order seasonal difference and first-order ordinary difference (Fig. 2). The preliminary judgment was as follows: D = 1 and d = 1. The autocorrelation function (AFC) and partial autocorrelation function (PAFC) plots of the first-order difference and first-order seasonal difference, respectively, revealed the following for the SARIMA model: p ≤ 1, q ≤ 4 for the number of exposures; p ≤ 3, q ≤ 2 for the number of cases; and p ≤ 1, q ≤ 2 for the number of hospitalizations (Fig. 3). Seasonal P and Q generally do not exceed 2nd order. A total of 16 models were finally constructed.

#### SARIMA model testing and evaluation

**Figure 4:**
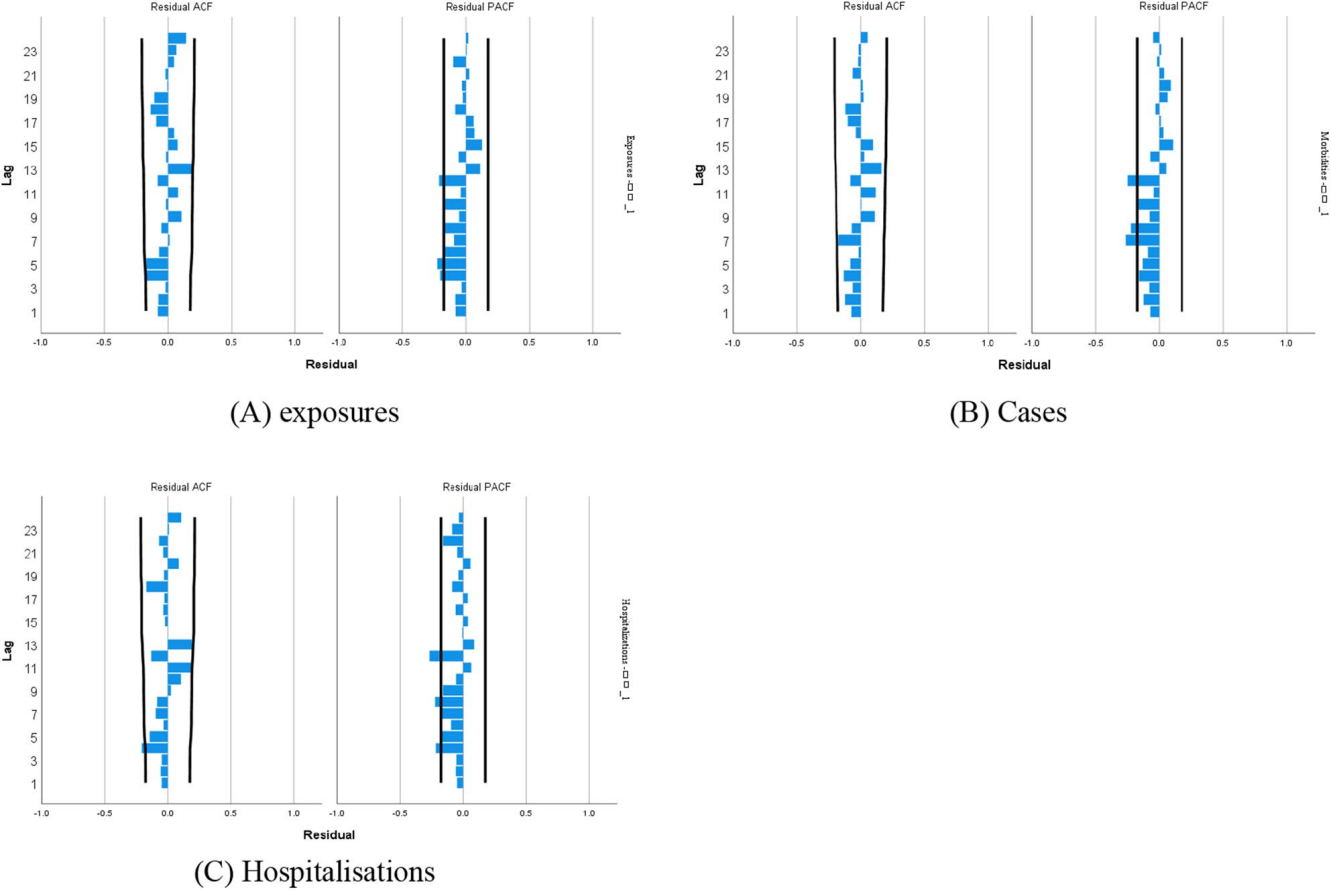
ACF and PACF function graphs for residual sequences.

**Figure 5:**
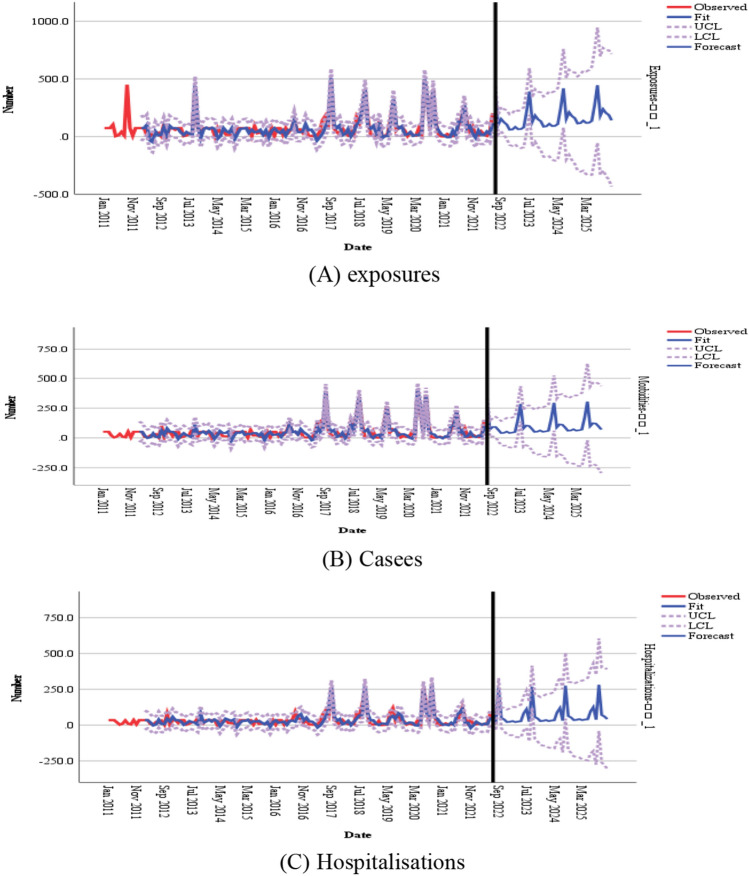
SARIMA (1, 1, 0) (0, 1, 1)_12_ trend prediction chart of mushroom poisoning outbreaks in Guizhou province.

After comparing the smoothed R, Root Mean Square Error (RMSE), Bayesian information criterion (BIC), and *P*-values of each model, the best model was finally selected: SARIMA_(number of exposures)_ (1, 1, 0) (0, 1, 1)_12_; SARIMA_(number of cases)_ (1, 1, 0) (0, 1, 1)_12_; SARIMA_(number of hospitalization)_ (1, 1, 0) (0, 1, 1)_12_ (Table 5). For three models, the LBQ test was used to calculate the white noise series of the prediction models, and *P* > 0.05, which conformed to the white noise series, indicated that the models were more adequate in extracting information from the data and the smooth R > 0.9 meant that the models fitted well. The residuals of the prediction models for the number of toxic mushroom poisoning exposures, cases, and hospitalizations were individually calculated, and the ACF and PACF plots of the residual series were constructed (Fig. 4). The ACF and PACF values were basically within the 95% confidence intervals, and the distribution of the residuals was random. The number of exposures, cases, and hospitalizations from January to June 2022 were within the prediction interval, combined with the prediction graph (Fig. 5). The actual values did not significantly differ from the predicted values; further, they were all within the prediction confidence intervals, demonstrating that the model prediction results had reference values.

**Table 5:** Model testing.

	SARIMA model	Smooth R	RMSE	BIC	*P*
Exposures	(1, 1, 0) (0, 1, 1)_12_	0.945	41.536	8.071	0.135
Cases	(1, 1, 0) (0, 1, 1)_12_	0.919	32.476	7.425	0.075
Hospitalizations	(1, 1, 0) (0, 1, 1)_12_	0.912	22.524	6.654	0.058

#### SARIMA model prediction results

The SARIMA (1, 1, 0) (0, 1, 1)_12_ model was used to predict the number of toxic mushroom poisoning outbreaks in Guizhou province from January 2022 to December 2025 in terms of exposures, cases, and hospitalization (Fig. 5). The prediction results suggested that there is no decreasing trend in the number of toxic mushroom poisoning outbreaks in 2025 in terms of exposures, cases, and hospitalizations compared with the previous years. The number of toxic mushroom poisoning outbreaks in terms of exposure, cases, and hospitalizations still showed a seasonal increase, with June–September being the peak period, in line with the characteristics of the previous years.

### Prophet model

**Figure 6:**
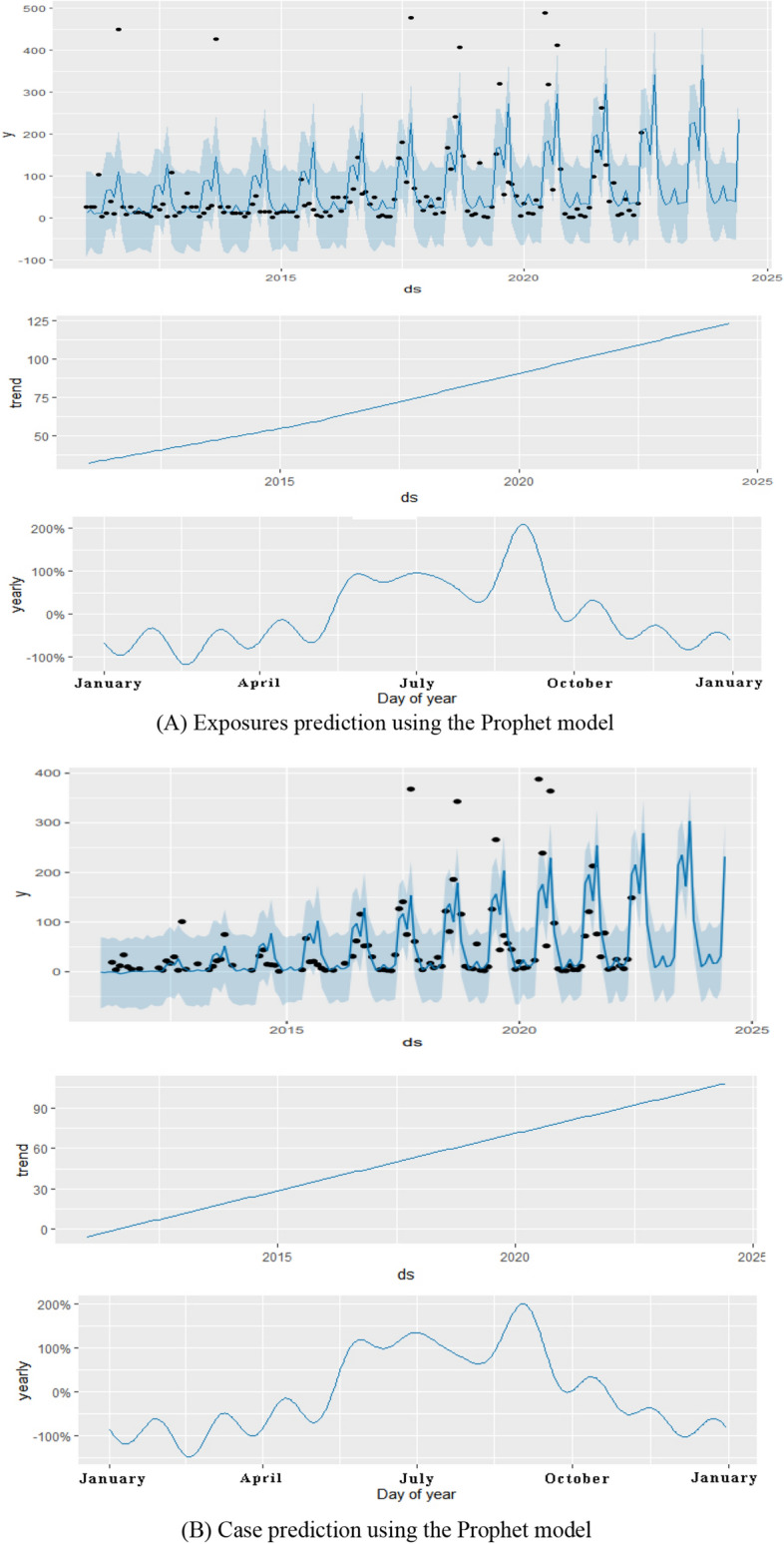

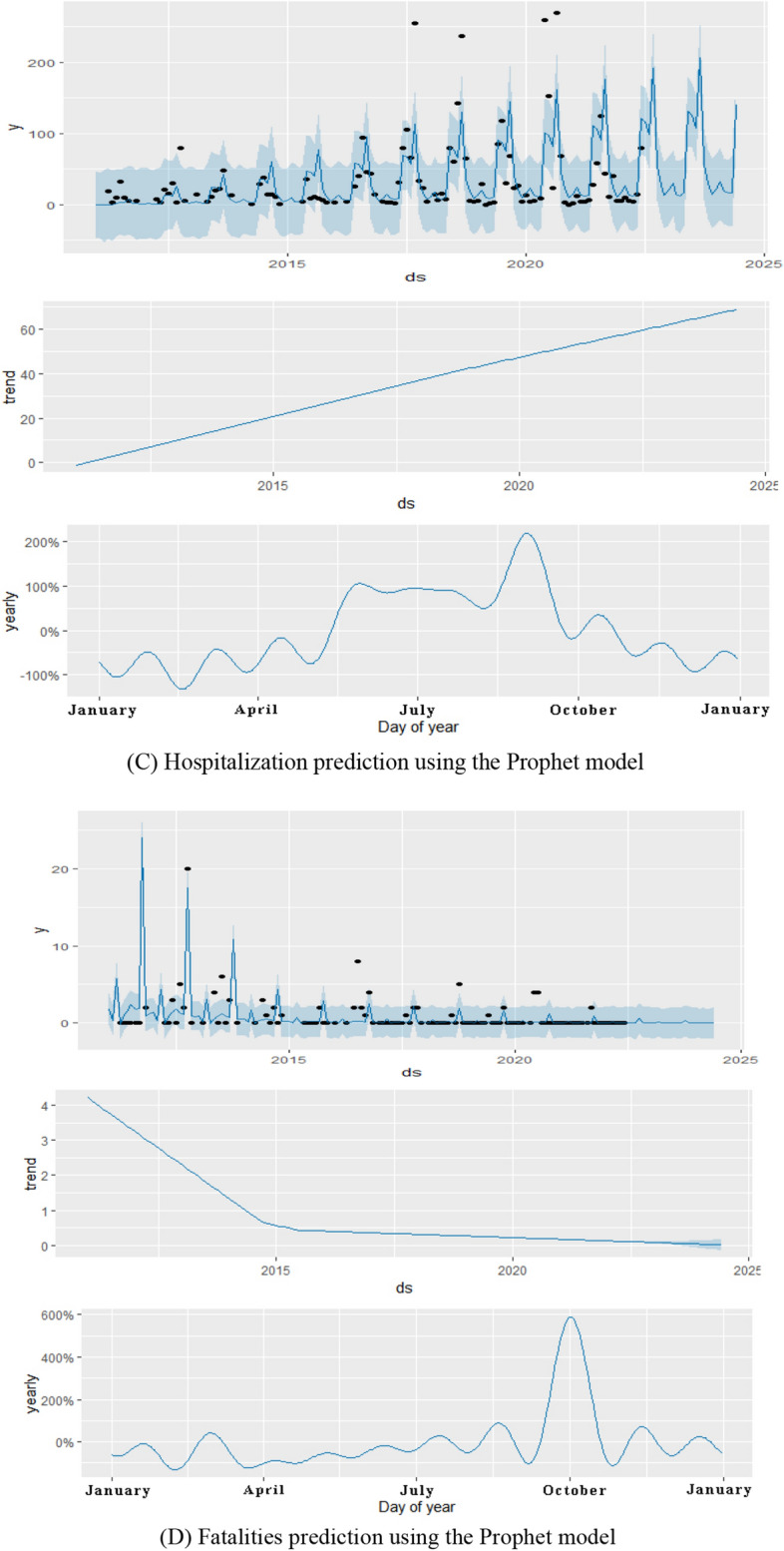
Prediction of mushroom poisoning outbreaks in Guizhou province using the Prophet model.

The Prohet package of R 4.2.0 software was used to construct a time series of mushroom poisoning outbreak data from 2012 to December 2021 in Guizhou province, and a Prophet model was established to fit and predict the data (Fig. 6). The evaluation parameters of the Prophet model were evaluated using the RMSE $\left ({X}_{RMSE}=\sqrt{\frac{{\sum }_{i=1}^{N}({X}_{obs,i}-{X}_{model,i}){ }^{2}}{N}} \right)$. The final parameter results are RMSE_exposures_ = 79.208, RMSE_patients_ = 47.638, RMSE_hospitalizations_ = 33.277, and RMSE_deaths_ = 2.270. As shown in the figure, the number of exposed, infected, and hospitalized individuals with mushroom poisoning in Guizhou province will continue to show an increasing trend in the future with significant seasonality, with obvious seasonality, mainly concentrated in June–September. Analysis of the survey report revealed that the places where the abnormal values in the graph occurred were mostly massive restaurants and rural banquets. From the predicted results, the number of deaths showed a clear downward trend; however, October was the peak month of the year when deaths were most likely to occur. We looked at the report for October and found that the main type of mushroom poisoning during this time was related to gooseberry mushrooms.

## Discussions

This study found that a total of 1577 outbreaks of mushroom poisoning occurred in Guizhou during this period, with a marked seasonal increase in the time of occurrence of the incidents, the rural household being the site of the largest number of outbreaks, and the overall mortality rate of people aged 1 ~ 6 years being higher than that of other age groups. Predictive models are widely used in various fields, but relatively few are used for early warning prediction of mushroom poisoning outbreaks. In this study, the prediction model is hoped to be used in the prevention and control work, which can provide a reference for the early warning and prevention and control work of mushroom poisoning. The two prediction models have better performance in predicting mushroom poisoning outbreaks in Guizhou Province, but each has its advantages and limitations.

Mushroom poisoning outbreaks in Guizhou province presented epidemiological characteristics that were consistent with most Chinese provinces^25^. Mushroom poisoning is the lead causative agent of foodborne pathologies and is on the rise^26^. The increase in reports of mushroom poisoning may be because the system for monitoring and reporting foodborne illnesses has become more effective^14^. The most noticeable increase in 2020 may have been caused because the majority of people in rural areas work outside their homes, and the government adopted a home quarantine policy in 2020 owing to the COVID-19 epidemic, which reduced the number of people going outside their homes and increased the opportunity for people to consume wild mushrooms^13,27,28^. Mushroom poisoning has noticeable seasonal and regional characteristics, with the season of abundant rainfall being the high-incidence period and rural households being the main place of occurrence, with the highest case fatality rate^29,30^. This may be because the environment, rainy season, and vegetation-rich areas in rural areas are more conducive to wild mushroom growth, resulting in increased chances of mushroom picking. In these areas, people are less aware of food safety and often seek medical attention only after the disease onset is severe; further, they are often far from hospitals with better medical resources, delaying the period of best treatment^31^.

The chi-square test revealed statistically significant variations in the incidence of poisonous mushroom poisoning in various cities and states, years, months, and incidence sites (*P* < 0.05). This suggests why different times and areas and climatic conditions, geographic environments, and human qualities all have a disproportionate effect on the possibility of mushroom poisoning. Further, it indicates that we should target our preventative and control efforts more precisely, taking into consideration regional circumstances^32^.

Based on the evaluation parameters RMSE and the graph of the prediction results from January to June 2022, we concluded that both models have relatively good predictive ability for mushroom poisoning outbreaks, although each model has strengths and limitations. The SARIMA model is better than the Prophet model (RMSE_SARIMA_ < RMSE_Prophet_) in predicting the number of exposures, cases, and hospitalizations. However, the Prophet model could be used to predict the number of deaths. The results of the Prophet model were more explanatory and could more accurately reflect the occurrence and seasonal trends of mushroom poisoning, as determined by the prediction plots of the two models. Nevertheless, the prediction results of the SARIMA model were more consistent with the actual results. The prediction outcomes of the two models were nearly identical. The Prophet and SARIMA models predicted an increase in exposures, cases, and hospitalizations, with the peak reporting period continuing to be the rainy season from June to September. Fortunately, there is a definite decreasing trend in the total number of fatalities, whereas seasonal patterns indicate that October is the month with the highest number of fatalities owing to the deadly fungal species *Amanita exitialis*^33,34^. The probability of death from mushroom poisoning is highly dependent on the type of mushroom; for example, highly toxic mushrooms of the *Amanita exitialis* cause acute liver damage and have a much higher mortality rate than other generally toxic mushrooms. This may be because although the time of high morbidity is the time when most generally toxic mushrooms are growing, the time of low morbidity coincides with the time when highly toxic mushrooms are growing, resulting in a lower mortality rate at peak morbidity than during periods of low morbidity. The combination of the two models could better predict trends in the occurrence of mushroom poisoning outbreaks for prevention and control efforts, as suggested by the prediction results. Nevertheless, all cities and states in the province should continue to focus on preventing the occurrence of mushroom poisoning. The results of the prediction model can be used for graded prevention and control, and dynamic risk grading can be managed for each area. The high-, medium-, and low-risk areas will be divided in each quarter, with emphasis on the monitoring and management of the high-incidence period and area. In addition, control measures and warnings should be based on factors such as the climate of the year, particularly the peak months of June–September, and medical institutions should pay considerable attention to mushroom poisoning cases in October.

Mushroom poisoning outbreaks occur due to a variety of factors, therefore this study is still deficient and did not include more influencing factors for prediction, and it is hoped that more influencing factors and other multivariate prediction models will be included in the future for more accurate prediction.

## Conclusion

Mushroom poisoning outbreaks maintain a high trend; therefore, it is important to strengthen predictive prevention and control efforts. Various prediction models can be combined with each other to meet specific needs in the prediction and control tasks, each with its own advantages. Further, the dynamic monitoring and reporting system should be implemented, with emphasis on the execution of a timely reporting system from the grassroots to higher levels of the health department and the development of precise and detailed epidemiological questionnaires on mushroom poisoning, clinical information sheets, and laboratory test result sheets to prepare the foundation for later studies on mushroom poisoning events and provide data support for dynamic adjustment of prevention and control policies and hospital clinical treatment strategies. Lastly, future propaganda should be based on the survey of local long-term residents on the knowledge of wild mushroom poisoning to fully understand the actual local situation in an easy-to-understand and enjoyable manner (such as a science animation video and propaganda animation manual) so as to conduct health activities and to ensure information is imbibed by each household.

## Contribution to the field statement

Mushroom poisoning has been recognized as a public health problem that threatens human health worldwide. In China, mushroom poisoning is the primary foodborne disease causative factor and the cause of the most related deaths. Mushroom poisoning has seriously endangered the life and health safety of the Chinese people. However, most of the current studies only describe past poisoning situations, and relevant prevention and control measures are proposed based on the data of previous occurrences. In this paper, we hope to change the previous passive prevention and control to active prevention and control by predicting the poisoning situation in advance, so as to provide a basis for reducing the harm of mushroom poisoning to people and formulating relevant prevention and control measures.

## Data Availability

Data for this study are available from the Public Health Surveillance Institute of the Guizhou Provincial Centre for Disease Control and Prevention. However, the availability of the data for this study is limited by the license used in the current study and therefore not publicly available. It can be obtained from the Guizhou Provincial Centre for Disease Control and Prevention (GPCD) under reasonable circumstances, and the right of interpretation rests with GPCD.
